# Delayed emergence of a global temperature response after emission mitigation

**DOI:** 10.1038/s41467-020-17001-1

**Published:** 2020-07-07

**Authors:** B. H. Samset, J. S. Fuglestvedt, M. T. Lund

**Affiliations:** CICERO Center for International Climate Research, P.b. 1129 Blindern, 0318 Oslo, Norway

**Keywords:** Atmospheric science, Climate change, Climate-change mitigation

## Abstract

A major step towards achieving the goals of the Paris agreement would be a measurable change in the evolution of global warming in response to mitigation of anthropogenic emissions. The inertia and internal variability of the climate system, however, will delay the emergence of a discernible response even to strong, sustained mitigation. Here, we investigate when we could expect a significant change in the evolution of global mean surface temperature after strong mitigation of individual climate forcers. Anthropogenic CO_2_ has the highest potential for a rapidly measurable influence, combined with long term benefits, but the required mitigation is very strong. Black Carbon (BC) mitigation could be rapidly discernible, but has a low net gain in the longer term. Methane mitigation combines rapid effects on surface temperature with long term effects. For other gases or aerosols, even fully removing anthropogenic emissions is unlikely to have a discernible impact before mid-century.

## Abstract (web summary)

Strong mitigation of anthropogenic emissions is necessary, but it is not clear how fast these efforts would lead to temperature changes. Here, the authors find that there is a substantial delay between reductions of emissions and a detectable change in surface temperature for a number of climate forcers.

## Introduction

This paper is about managing our expectations. If we were to strongly mitigate emissions of one particular climate-altering gas or aerosol today, potentially tying up vast amounts of resources and public good will, when would we reap the benefits in terms of reduced levels of climate change? The answer to this question is highly non-trivial, partly because natural variability strongly affects trends on decadal scales, and partly because once mitigation has been put in place, we no longer know what the climate would have been if we had not.

Current observed climate change is primarily the net result of a range of anthropogenic emissions and other physical changes to the global environment^1,2^. Since the 1970s, this anthropogenic forcing has resulted in increased global mean surface temperature (GMST) at a rate of on average 0.2 °C per decade^3^, and most future projections see this overall evolution continuing for several decades regardless of emission scenario^4^. Achieving the aims of the Paris Agreement, however, requires substantial and rapid mitigation across a range of climate forcing emissions—potentially entailing substantial costs and short-term perceived burdens on society. For climate mitigation efforts to maintain public support, it is therefore likely crucial to be able to document the benefits. While changes in the growth rates of atmospheric concentrations of greenhouse gases might be more readily discernible^5^, the central indicator of progress would be a reduction in the rate of surface warming relative to what is anticipated under some assumed baseline emission scenario (or, in practice, the rate observed over the last decades).

On annual-to-decadal scales, this rate of warming is however substantially affected by the interplay between anthropogenic forcing and internal variability. The so-called hiatus period of 1998–2015 is a good illustration, where most indicators of climate change continued to evolve while global mean surface temperature had a reduced rate of increase^6^. The question facing us is therefore how to determine that progress has been made towards the ambitions of the Paris Agreement, and that this is a consequence of changes in anthropogenic influence on the climate. Such emergence of a climate mitigation signal beyond natural variability can never be proven, as we would be comparing to an unknown, counterfactual world. It is, however, possible to be clear about our expectations, based on the currently best available science, and then to evaluate future climate observations relative to this.

Previously, Tebaldi and Friedlingstein^7^ (hereafter TF13) have quantified the expected delayed detection of climate mitigation benefits due to climate inertia and variability. They found that for global mean surface temperature, emergence would occur ~25–30 years after a heavily mitigated emission pathway (RCP2.6) departs from the higher ones (RCP8.5 or RCP4.5). At the time of writing, that translated into 2035–2045, where the delay was mostly due to the impacts of the around 0.2 °C of natural, interannual variability of global mean surface air temperature (GSAT, see “Methods”), and the general inertia of a climate system out of equilibrium. They also showed that for smaller (but more policy and societally relevant) regions, where natural variability is intrinsically higher, the detection time occurs a decade or more later.

More recently, Marotzke^8^ (hereafter M18) investigated the range of near-term warming rates under very strong climate mitigation (RCP2.6), and found that in over a third of 100 realizations (members of an initial condition ensemble, i.e. identically forced simulations differing only by internal variability), the world would still warm faster until 2035 than it has done for the past two decades (i.e. a higher 15-year trend for 2021–2035 than for 2006–2020). He warns that we might face what they term a hiatus debate in reverse, where the most well-known indicator of climate change (global mean surface temperature, or GMST; see methods for the distinction between GSAT and GMST) continues to rise even after massive, international efforts to mitigate emissions. This might, in turn, present a substantial challenge for communication and science-policy interactions.

The key result of both TF13 and M18 is that we should not expect immediately measurable impacts on global mean temperature evolution, even under very substantial mitigation. They put their main emphasis on mitigation of CO_2_, which is the main driver of both historical and future anthropogenic climate change, or take a scenario approach where a whole basket of emissions are mitigated simultaneously. However, as some key anthropogenic emissions can in principle be mitigated separately from CO_2_—and with different costs per tonne of avoided emissions^9,10^—it is crucial to also investigate the time of emergence of a detectable, significant change relative to a higher emission scenario from curbing emissions of these components.

In this paper, we extend the work of TF13 and M18 to cover mitigation of individual climate forcer (or precursor) emissions, by combining reduced complexity modelling with a large, single-model initial condition Earth System Model (ESM) ensemble to to account for internal variability. Our key finding is that for the majority of current anthropogenic climate forcing (and precursor) emission types, including CO_2_, CH_4_, N_2_O, aerosol species, and a range of other gases, a significant change in surface temperature evolution in response to even very strong mitigation policy will not occur until decades after efforts are put in place. We investigate both cumulative differences with respect to a baseline scenario, and potential near-term changes to the rate of global mean surface warming. Combined mitigation of multiple components, as envisaged under most climate scenarios, may result in more rapid emergence, but will also imply offsetting between warming and cooling effects. Even fully removing anthropogenic emissions of warming short-lived climate forcers, such as black carbon, in isolation, would not be discernible with statistical significance for a decade.

## Results

### Defining emergence

Our core methodology is to simulate the evolution of global mean surface temperature, under a given emission pathway, using the reduced-complexity climate model MAGICC6^11^, and combining this with internal variability extracted from the CESM1 Large Ensemble (LENS)^12^. Emergence of a climate signal is then defined as the time when the surface temperature change resulting from following one pathway would differ in a statistically significant way from another, in which emissions of one or more components have been mitigated, when taking variability into account. Note that despite similar terminology, this is a distinct question from quantifying the emergence time of a climate state different to that of the recent past, as has been the focus of another recent set of studies^13–15^. Below, we first apply our analysis to the full RCP pathways, to estimate the time-of-emergence for different scenarios to be distinguishable from one another. Then we discuss the temperature impact of three idealized mitigation scenarios, in 2100, before showing how our combination of reduced complexity modelling and internal variability looks for one example forcer. We then estimate the emergence time for each combination of scenario and forcer (see below), before discussing which forcers yield the most rapid, significant reductions in surface temperature relative to a future where our emissions follow RCP4.5.

The term emergence requires a strict definition. TF13 asks by what time two differently forced simulations of annual mean surface temperature differ by more than the internal variability, and quantify emergence as the time when a significant trend can be found in the difference between two simulations. M18 takes a more generalized approach based on Bayesian statistics, and quantifies the probability that a given change (e.g. a policy driven change in emissions) is sufficient and/or necessary to cause an observed difference in trends in surface temperature. (See M18 for a thorough review of other recent uses of the emergence concept.).

While we document consistency of our results with both trend-based approaches below, we here take a third approach. Consider a year in the future, say 2050. At that time, we will have had 30 measurements of annual mean temperature since 2021. For a pair of simulations, with and without a mitigation policy applied (in our case RCP4.5 and a case with an idealized mitigation scenario applied to a single component), we ask whether these 30 measurements are significantly different according to a Student’s *t*-test (*p* < 0.05). More generally, the first year after 2021 where this is true is defined as the time of emergence for that particular simulation pair. Full emergence is subsequently defined as the first year when at least 66% of the constructed ensemble members show such significant difference from the baseline (RCP4.5) when taking into account the period from 2021 and up to that year. This approach utilizes all observations that would be available at a given time after a hypothetical mitigation effort, and in our case has the advantage of being able to detect the potential rapid impact of idealized step perturbations without relying on a trend calculation. That said, as we show below, trend-based calculations following TF13 and M18 do give consistent results where comparable.

### Emergence from multi-component mitigation pathways

**Fig. 1:**
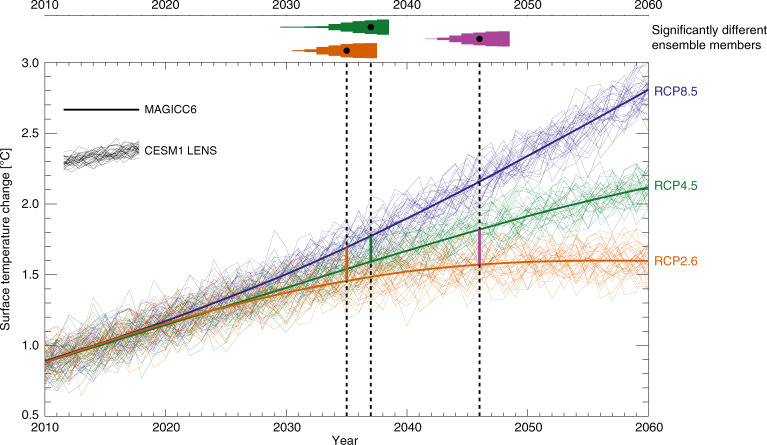
Surface temperature response to multi-component mitigation. Emergence of a temperature signal when going from one RCP emissions pathway to another. Thick lines show calculations from MAGICC6. Thin lines show evolutions of interannual variability of global mean surface temperature, extracted from the CESM1 LENS. Triangles above the graphs indicate the number of ensemble members where the temperature difference between the RCPs is significant, when accounting for internal variability.

In Fig. 1, we show how MAGICC6, in its default configuration (see Methods), calculates global mean surface temperature when following RCP8.5, RCP4.5 and RCP2.6. Visually, RCP8.5 starts diverging from the others already in 2020, while RCP4.5 and RCP2.6 start differing around 2025 (see the thick, smooth lines). When adding on the internal variability from 32 CESM1 LENS ensemble members, however, the pathways overlap until much further into the 21^st^ century. Above, the triangular shapes illustrate the number of ensemble members that are significantly different (as defined in Methods, with an integration time starting in 2011) for a given year, with the black dots denoting 66% (21) of the members. For a situation where we take RCP8.5 as a reference, and wish to estimate when we would expect a quantifiable effect of instead following RCP2.6, we find 2035 to be the year of emergence. Following RCP4.5 rather than RCP8.5 would have been visible, for 66% or more members, from 2037. Going from RCP4.5 to RCP2.6, however, which are both markedly lower emission pathways than RCP8.5, can only be expected to be visible from 2046.

These results are consistent with a similar analysis in TF13, and lend confidence to our definitions and method of calculating significance and emergence. We also check consistency with M18 who used a somewhat different approach, investigating the probability of two consecutive 15-year GSAT trends being different under RCP4.5 and RCP2.6 emissions, respectively. M18 used the MPI Grand Ensemble^16^, and calculated a 45% probability of the 2021–2035 trend being lower than 2006–2020 in RCP4.5, and 67% in RCP2.6. I.e. the lower emissions seem to increase the probability of a trend reduction. M18 also report a 22% probability that this shift was caused by the difference in emissions, in a necessary and sufficient sense, according to Bayesian statistics. Redoing their analysis for the CESM1 LENS, we find 59% and 80% probabilities of a lower GSAT trend for 2021–2035 than 2006–2020, in RCP4.5 and RCP2.6 respectively, and a 20% probability of (necessary and sufficient) causation. Similar overall consistency was found for the 2036–2050 period. (For details of this method, see M18.)

### Emergence from mitigation of single climate forcers

From here on, we discuss idealized mitigation of single climate forcers, following the scenarios defined in Table 1. Most industrial activities co-emit a broad range of species, but often with distinctly different composition across individual sectors and sources^17^. Hence, in reality, targeted mitigation of a single species would often be difficult. However, their climate forcing (and subsequent temperature response) is commonly thought of as independent and linearly additive^2,18^, enabling co-emission analyses based on single component results. Moreover, exceptions do exist. One notable example is methane from the production and distribution stage of the energy sector, which can largely be mitigated independently from the sector’s combustion-related emissions of SO_x_ and CO_2_. Methane would also be the species primarily affected by measures directed at the agriculture and waste management sectors^19–21^. While a detailed discussion of the feasibility of targeted mitigation of a single species is beyond the scope of the present-study, Supplementary Fig. 1 shows the interconnected nature of near-present day emissions (2014 numbers) by breaking down the major economic sectors by component.

**Table 1:** Idealized emission scenarios.

Emission scenario	Description
Zero emissions	Emissions of one climate forcer set to zero, starting to 2020. Other components evolve according to RCP4.5.
−5 % per year	As Zero emissions, except that emissions of that forcer are gradually reduced by 5% per year, starting in 2020.
RCP2.6	As Zero emissions, except that emissions of that forcer start following RCP2.6 in 2020.

Each of these three scenarios is applied for each climate forcer considered; see list in Table 2.

Table 2 shows the emission and climate forcing components we consider, and the temperature implications of our mitigation scenarios in 2100, relative to a world where emissions follow RCP4.5, as calculated by MAGICC6 with default tuning and an Equilibrium Climate Sensitivity of 3 °C. These numbers can be seen as avoided warming resulting from our assumed, idealized mitigation (or additional warming, in the case of negative numbers). Our key question, however, is whether we could expect to observe this difference in actual temperature measurements before the end of the century. Bold numbers in Table 2 indicate that the year of emergence occurs prior to 2100 for that component/scenario combination. As examples, making CO_2_ (alone) follow RCP2.6, rather than RCP4.5, would result in 0.74 °C of avoided global warming in 2100. Fully mitigating anthropogenic emissions of black carbon (BC), however, would avoid only 0.09 °C, consistent with recent multi-model results^22,23^. Combined zeroing of emissions of the three major anthropogenic aerosol species (BC, organic carbon (OC) and sulphate precursors (SO_x_)) would, however, give a net effect of 0.16 °C of additional warming, due to the loss of cooling from aerosol scattering. This result is markedly lower than what was recently found in an idealized study using four ESMs^24^, indicating that there are responses in the complex models that may not be fully captured in the version of MAGICC used in the present study. One potential reason is that its response is tuned using the output of CMIP3 models, which had poorer representation of the aerosol-cloud interaction than is present in the current generation of ESMs. Another is the lack of regionally resolved aerosol-cloud interactions, and their projections onto teleconnections and modes of variability^25^.

**Table 2:** Global temperature implications.

Forcing species	Zero emissions	−5 % per year	RCP2.6
CO_2_	**1,05**	**0,74**	**0,74**
CH_4_	**0,19**	**0,16**	**0,17**
N_2_O	**0,18**	**0,14**	0,06
SO_x_	**−0,13**	**−0,09**	**−0,09**
CO	0,02	0,01	0,00
NMVOC	**0,04**	**0,04**	0,01
NO_x_	**−0,08**	**−0,06**	0,00
BC	**0,09**	**0,07**	**0,04**
OC	**−0,12**	**−0,10**	**0,07**
CF_4_	0,01	0,01	0,01
SF_6_	0,01	0,01	0,01

Avoided warming in 2100, when implementing idealized mitigation relative to RCP4.5. All numbers are in °C. Negative values imply global warming above RCP4.5, due to the removal of presently cooling emissions. Numbers in bold are significant in 66% or more of the ensemble members in the subsequent emergence analysis, i.e. that time-of-emergence occurs before 2100. All numbers are calculated with MAGICC6, with an assumed equilibrium climate sensitivity of 3 °C.

**Fig. 2:**
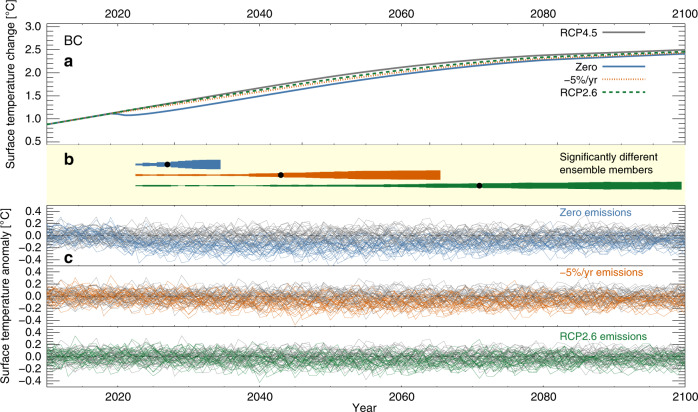
Single component mitigation. Example of the emergence of a temperature signal when mitigating one climate forcer. **a** MAGICC6 calculations following RCP4.5 (black line), and then modified by mitigating BC emissions according to one of three idealized scenarios starting in 2020 (zero emissions, −5% per year, or switching to RCP2.6). **b** The number of ensemble members where the temperature time series, from 2020 and up to that year, is significantly different from zero (*t*-test, *p* < 0.05). Black dot: 21 significant ensemble members (66%). **c** The (detrended) temperature difference when variability from CESM1 LENS is taken into account.

In Fig. 2, we illustrate our method and calculations for the case of BC. Panel (a) shows the idealized surface temperature calculations from MAGICC6, and how the three reduced emission pathways would differ from RCP4.5. BC, which has an atmospheric lifetime of only a few days due to efficient wet scavenging^26^, is rapidly removed, resulting in a rapid change to the surface temperature already in the first years. However, even for the case of zero anthropogenic emissions, the bands that span the internal variability (panel c) do not fully separate. Also, we see that the difference is clearest for the period 2025-2050, after which the BC emissions in RCP4.5 also start declining, bringing the two evolutions closer together. In panel (b), we again show how the number of ensemble member pairs with significant difference increases. A signal from zeroing of anthropogenic BC would emerge already in 2027, while transitioning BC emissions from RCP4.5 to RCP2.6 from 2020 and onwards would only be clear from around 2070.

**Fig. 3:**
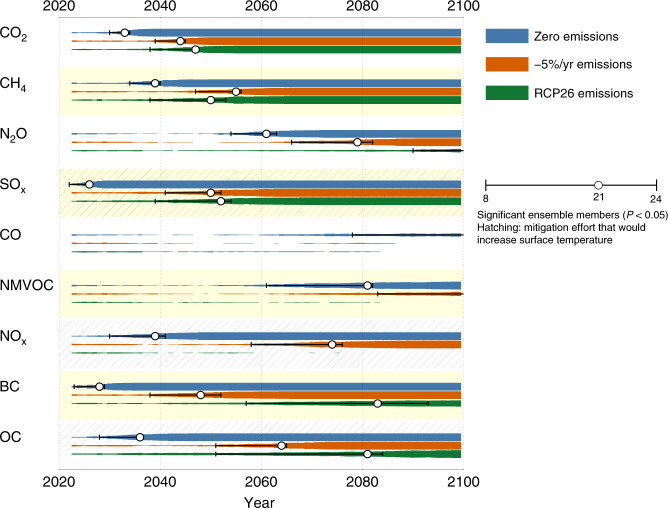
Emergence times after single forcer mitigation. Time of emergence of a global mean surface temperature signal for idealized individual mitigation efforts of a range of short- and long lived climate forcers. The colored expanding bars show the evolution of a statistically significant signal (*t*-test, *p* < 0.05), from zero (minimum) to 32 (maximum) ensemble members. The circles show the first year when 66% (21 members) show significant signals. The error bars show the 25–75% range (8 and 24 significant members respectively). The underlying calculations are illustrated in Fig. 2. Hatching indicates a positive global temperature change in response to the mitigation (i.e. loss of cooling).

Figure 3 shows the results of the corresponding analysis for all our considered forcers and scenarios. Some emissions, like CO_2_ and CH_4_, can be expected to have an early impact on surface temperatures due to the amount of emissions and strength of their current forcing, while others, like CO that acts indirectly on the climate via changes to ozone and methane levels, would not have a measurable impact on global mean surface temperature in this century—even for zero anthropogenic emissions from 2020. This does not mean that such mitigation is wasted, as the impacts would combine with, and strengthen, the effects of other aims such as air quality improvement or pollution reduction. It does, however, clearly illustrate the well-known fact that some forcers presently do not have a very significant effect on the global mean temperature relative to internal variability. Table 3 lists the years of emergence for each combination (excluding species where no emergence is found). We discuss these individual forcer results further below. Note that the sign of the temperature change is not readily visible in Fig. 3. For example the early emergence of the effects of SO_x_ mitigation, for all three scenarios, all lead to additional rather than avoided warming (see Table 2). Hatched background indicates the cases that lead to additional warming.

**Table 3:** Emergence times.

Forcing species	Zero emissions	−5 % per year	RCP2.6
CO_2_	2033	2044	2047
CH_4_	2039	2055	2050
N_2_O	2061	2079	–
SO_x_	2026	2050	2052
NMVOC	2081	–	–
NO_x_	2039	2075	–
BC	2028	2048	2083
OC	2036	2064	2081
CH_4_ (ECS = 2)	2042	2060	2057
CH_4_ (ECS = 4)	2038	2052	2048

Year of emergence, after mitigation of one climate forcing component from 2020, defined as the year when half or more of the ensemble members are significantly different from the baseline (RCP4.5) according to a Student’s *t*-test. (See “Methods”).

### Mitigation influence on the rate of surface warming

**Fig. 4:**
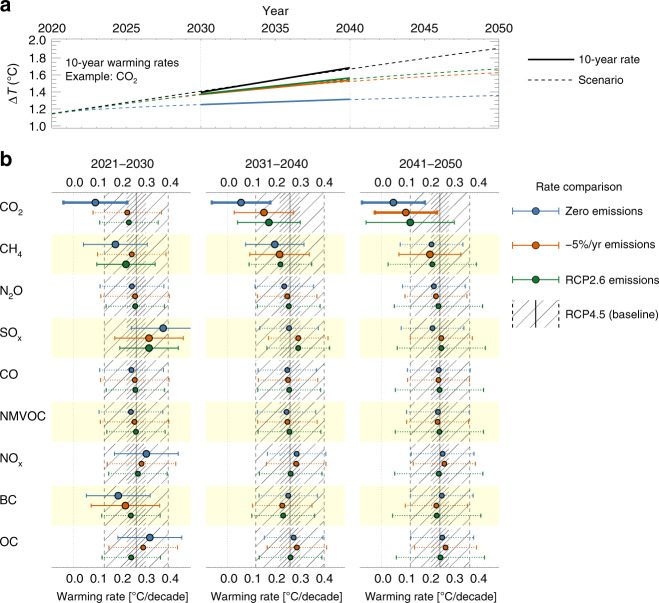
The impact of single component mitigation on rates of warming. **a** Example calculation for the CO_2_ mitigation scenarios (dashed lines). The solid lines show the 10-year average trends for 2031-2040. **b** Warming rates for three coming decades (columns), for each component and scenario (colored bar-and-whiskers), compared to RCP4.5 (black lines and hatching). Projections calculated with MAGICC6, internal variability is taken from CESM1 LENS (32 ensemble members). Dots and whiskers show the mean and ±1 standard deviation of the rates in the mitigated scenarios. Solid lines indicate where the mean trend is outside a third of the standard deviation of trends from LENS evolution added to RCP4.5 (dense hatching). Large symbols and fat lines show when the trend is outside one standard deviation in RCP4.5 (open hatching).

Another common quantity in discussions of the evolution of climate change is the rate of surface warming. Since the 1970s, we have observed an average rate of 0.2 °C per decade, according to recent assessments^3^. In Fig. 4, we analyse the rate of global mean surface temperature change in the three coming decades, under RCP4.5 combined with internal variability from CESM1 LENS (32 ensemble members), and under each of our scenarios of individual component mitigation (also 32 members in each case.) We note that MAGICC6 projects a somewhat higher rate of warming than observed, at around 0.25 °C per decade. This is a feature of this particular model^27^, and we do not discuss it further other than to note that it forms the baseline for the following argument.

For the mitigation scenarios, Fig. 4 indicates that whichever single climate forcer or emission is mitigated, one should not expect to see strong impacts on the rate of warming before mid-century. The clearest exception is if emissions of anthropogenic CO_2_ were completely removed. This would drop the rate of warming to outside one standard deviation of the rate in RCP4.5 already in the first decade (2021–2030), and as CO_2_ is also the main future driver of warming in the base scenario the rate would remain low. Figure 4 also illustrates the difference between long and short-lived climate forcers. Note BC and methane, where strong mitigation would bring the mean rate across the ensemble members below the baseline mean in the first decade, but then back in line with RCP4.5 in later decades as CO_2_ emissions again become dominant. The same is true for SO_x_ mitigation, which however would rather increase the rate of warming in the near term^28^. (Note that SO_x_ is rarely emitted in isolation, so co-emission would likely alter this result^24,29^).

Overall there remains a marked chance that whatever single-component mitigation is implemented, future decades with warming rates at or above the present (as represented by MAGICC6) would be expected. The situation could be different if several components were reduced in consort, through a combined mitigation strategy such as sector-based policy; see Discussion, and also the analysis of the full RCP pathways presented earlier. In Fig. 4 we showed 10-year trends. Supplementary Figure 4 shows a similar analysis for 15-year trends. While the spread between ensemble members is clearly smaller when allowing for a longer period of integration, the number of components or scenarios with signals that emerge is not markedly different.

### Mitigation potential versus absolute emissions

**Fig. 5:**
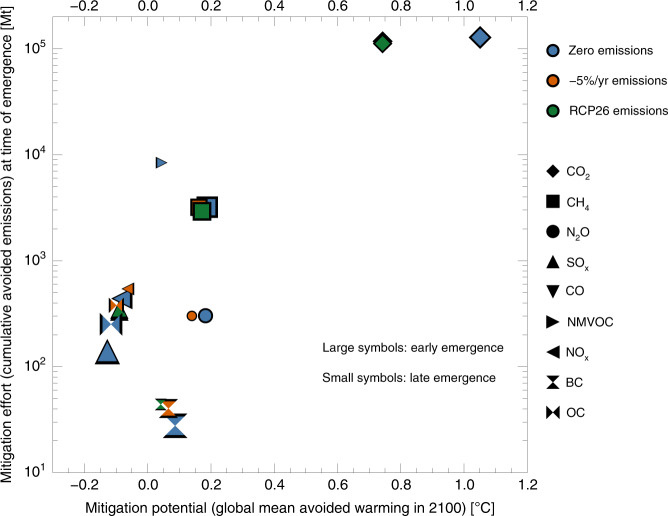
Potential versus effort for single forcer mitigation. Mitigation potential (avoided temperature increase in 2100) versus mitigation effort, here quantified through the total mass of mitigated emissions at time-of-emergence. Global mean surface temperature change in 2100 was calculated using MAGICC6. The symbol size scales with time-of-emergence, large symbols indicate early emergence, small symbols later emergence. See Table 3 for time-of-emergence numbers.

Finally, in Fig. 5, we plot the mitigation potential of a single emission component versus the effort it requires to achieve emergence of a mitigation signal—i.e. which component can give the most bang for the buck. We quantify the latter as the total amount of mass that is mitigated (emissions avoided) at time-of-emergence. This gives a first order indication of the amount of effort required to implement our idealized mitigation scenarios. The figure correlates surface temperature change in 2100 (relative to RCP4.5) with the cumulative mitigated mass (in megatons; see the sidebar for the precise units). The size of the symbols scales with the time of emergence, with large symbols showing earlier emergence times. Our analysis confirms that for mitigation to be rapidly effective at reducing the rate of global warming, targeting BC emissions (where they are not co-emitted e.g. with SO_2_) would be efficient—however, with a low final payoff. CO_2_, on the other hand, has the largest payoff, but also requires more than three orders of magnitude more mitigation in terms of mass before the effects can be expected to emerge. Methane can be seen as a middle-of-the-road species if choosing between targeted mitigation policies, with relatively rapid emergence, and several tenths of a degree of potential avoided warming by 2100.

## Discussion

At an overall level, our results show that even if the global temperature response to mitigation of one type of anthropogenic emission can readily be calculated using emission metrics^30,31^ and simplified modelling^11,32^, internal variability will preclude the rapid emergence of a discernible signal for plausible mitigation pathways. This is in line with previous studies looking at combinations of mitigation measures^7,8^. Other effects, such as feedbacks through excitation of modes of variability, geophysical processes not properly treated in the underlying models, and events such as volcanic eruptions, may add further complications to such a detection^33^. Hence, our results should be seen as a lower limit—and, further, founded on the details of the scenarios and models we have employed.

One clear caveat for our study is the reliance on the year-to-year variability calculated by CESM1 LENS, under the assumption of RCP8.5 emissions. The representation of variability on global temperature is known to differ significantly between CMIP5 generation models^34^, and while the overall performance of the LENS has been evaluated against observations^12^ we can here only say that it is not a clear outlier in the multi-model ensemble^34^. However, for the sake of the present analysis we have confirmed that the variability does not change between 2020 and 2100, when calculated across the whole ensemble A further caveat is the reliance on the responses and parameterizations of MAGICC6, including the assumption of an Equilibrium Climate Sensitivity (ECS) of 3 °C. Recent work has shown that near-term surface warming rates in MAGICC6 are higher than in a comparable model (FaIR)^27^, a difference that indicates that our results might have been different had we used another simplified climate model—in particular for the discussion of rates of warming over the next decades (Fig. 4). However, given the modest size of the response to most of our perturbations, this is unlikely to have major implications for the overall results.

The carbon cycle treatment in MAGICC6 is also worth noting. We have opted to use the default parameterization, which is based on the Bern model contribution to C4MIP^35^. Supplementary Figure 3 shows the CO_2_ and CH_4_ concentrations projected by MAGICC6 for the RCP scenarios, and from our perturbations to these two components applied after year 2020—indicative of the proportion of emissions that remains in the atmosphere after carbon cycle calculations. We also show observed global, annual mean values from NOAA ESRL. TF13 showed that the interannual variability in global mean greenhouse gas concentrations is so low that a signal rapidly emerges when transitioning from one RCP to another. This is also indicated in our figure, where variability in the observations (red line) is not much larger than in the MAGICC6 simulations. Further, we can see that for CO_2_, a 5% decrease per year from 2020, and transitioning to RCP2.6 emissions in 2020, both yield very similar concentrations to a situation where RCP2.6 was followed since 2005 (where the pathways starts). A zeroing of anthropogenic emissions in 2020, however, yields markedly lower concentrations. This difference persists through the century. For CH_4_, we first note that MAGICC6 projects slightly lower concentrations than what is observed from 1990 and on. This may influence the estimated temperature mitigation potential of CH_4_, as shown above. Also, we note that the concentrations resulting from a 5% decrease per year from 2020, and from transitioning to RCP2.6 emissions in 2020, are initially different but converge with the overall RCP2.6 evolution already in 2045. All pathways result in the same CH_4_ concentration in 2100, of around 1200 ppb.

A further point of note is that our perturbations were applied to the RCP4.5 pathway in 2020. At this time, some reductions in short lived forcer emissions have already been assumed to have taken place^29,36^. This has two implications. Firstly, it introduces a dependence in our results on the starting year for our idealized scenarios. A similar analysis started e.g. in 2000 would likely have shown stronger deviations from the baseline and hence an earlier emergence. The second implication is that our starting state does not necessarily correspond to the actual global emissions of 2020, as no harmonization of RCP4.5 emissions with more recent observations has been done. One example is SO_2_ emissions in China, which have dropped more strongly than assumed in RCP4.5^37^. These factors may influence the numerical values of our results, but are unlikely to affect our overall conclusions.

One last question is whether our results can be taken to be additive, and used e.g. to study the impacts of sector-wide policy and co-emission of multiple species. While emergence years from single-component mitigation scenarios cannot be added directly, our underlying framework could readily be combined in this way. As a sensitivity test, we performed one additional simulation where all our treated components were set to RCP2.6 emissions from 2020, i.e. the sum of all our RCP2.6 perturbations. The resulting temperature change in 2100 is 0.99 °C, while summing the relevant results in Table 2 yields 1.04 °C. This difference is within the numerical uncertainty of our analysis. We note, however, that in realistic situations there will be biogeophysical effects (e.g. interactions between the components, geographically unevenly distributed feedbacks, nonlinearities in aerosol-cloud interactions) that are not captured in a simplified, linearized model such as MAGICC6, and would require computationally much heavier simulations with comprehensive Earth System Models. The advantage of this extra layer of complexity is that it would also allow for studies of regional patterns, and changes in other societally relevant variables such as precipitation and extreme events. Our results can be used to guide the development of such model experiments – and to look for situations where such interactions or nonlinearities are especially prominent.

Rigorous detection and attribution of the impacts of even very strong mitigation efforts on global mean surface temperature will, for a long time, be challenging. Such mitigation is, however, crucial in order to achieve the aims of the Paris Agreement. It is therefore imperative that the scientific community explores and clearly communicates the expectations we have in terms of quantifiable, observable impacts. Here, we have shown that for the majority of the components of our net climate impact, any emergence of a significant change in surface temperature—relative to a higher-than-realized emission scenario—will not occur until decades after efforts are put in place.

The most rapid, significant climate change mitigating impacts of emission reductions would come from heavy mitigation of CO_2_, CH_4_ or BC—fully consistent with previous literature. These efforts could all be visible by mid-century, but likely not before. Rates of warming cannot, however, be guaranteed to drop below the present rate in any given decade, as the near-term influence of internal variability is very strong. The effect from mitigation of sulphate aerosols would also be detectable early, but would drive temperatures up rather than down due to loss of present cooling. Other emissions, such as NO_x_, organic carbon and non-methane volatile organic components, would likely not have a significant impact on global mean temperature until the second half of the century, even for very heavy mitigation. Concurrent, multi-component mitigation, however, as assumed in the lower RCP scenarios (or the Shared Socioeconomic Pathways), has the potential to be detectable around 2040. These are expectations that need to be clearly explained and communicated to policy makers, and to the public, if we wish to avoid a backlash against perceived ineffective mitigation policies. Consequently, other indicators of progress towards the Paris agreement, such as concentrations or anthropogenic emissions of greenhouse gases or the carbon intensity of the global economy are key variables to focus on—at least for the coming decades.

## Methods

### Emission mitigation scenarios

The basic methodology of this paper is to simulate the future climate evolution under a set of idealized emission mitigation scenarios, using the MAGICC6 simple climate model and accounting for internal variability by adding results from the CESM1 Large Ensemble, and calculate if and when a signal, in terms of global mean surface temperature change, emerges from the noise.

All results shown in this paper are based on climate models. Hence, global mean surface temperature is defined as the air temperature at a reference height of 2 meters (GSAT, or global surface air temperature). We note that this definition is subtly different from the global mean surface temperature (GMST) definition usually used for observational time series over the historical era, which blends surface air and sea surface temperatures. For our purposes, this distinction does not affect the overall results.

All our emission pathways start with one of the Representative Concentration Pathways (RCPs)^36^, adopted by the IPCC for its fifth Assessment Report (AR5) and still in broad use today. As the baseline for most of our mitigation scenarios we use RCP4.5, which was assessed in the AR5 to lead to a 2.4 (1.7–3.2) °C global mean surface temperature increase by the end of the century, relative to 1850-1900^38^. Additionally, we use RCP8.5 (strong emissions increase) and RCP2.6 (strong mitigation, and broadly consistent with the Paris agreement) as illustrations.

As mitigation scenarios, we individually consider three idealized cases, for each of a range of climate forcers; see Table 1. Our three cases involve either a zeroing of anthropogenic emissions of one component from 2020 and onwards (relative to RCP4.5), a 5% reduction per year from 2020, and a switch in 2020 from RCP4.5 to RCP2.6. Each of the resulting emission pathways is then run through MAGICC6. Table 2 lists the forcing components we consider, as well as the changes in global mean surface temperature calculated by MAGICC6 in 2100, relative to RCP4.5. Emissions are shown in Supplementary Fig. 2.

### Reduced complecity simulations with MAGICC6

MAGICC6 is a climate model of reduced complexity, built around an energy balance equation. It contains a hemispherically averaged upwelling diffusion ocean coupled to an atmosphere layer, and a globally averaged carbon cycle model^11^. It takes as input time series of annual emissions of most major anthropogenic climate drivers, converts them to atmospheric concentrations via the carbon cycle or other parameterizations of lifetime and removal, then calculates radiative forcing via preset forcing efficiencies. From there, it calculates surface temperature responses via an assumed climate sensitivity and per-species efficacies. Its multitude of settable parameters have been pre-tuned to match the response of more complex Earth System Models, such as the CMIP3 ensemble (model default). In the present paper we run MAGICC6 in its default configuration^11^. This further includes an assumed Equilibrium Climate Sensitivity of 3 °C, which has long been the centre point of scientific assessments^39^, and a carbon cycle response based on the Bern model contribution to C4MIP^35^. Efficacies are 1 for most species, except the aerosol radiation interaction which has an efficacy of 0.9.

### Internal variability from CESM1 LENS

To add an estimate of internal variability to the temperature evolutions from MAGICC6, we use the CESM1 Large Ensemble. Here, the CESM1 Earth System Model was run multiple times with a combination of historical and RCP8.5 emissions, differing only by the initial conditions in 1920. The result is an ensemble of 32 climate simulations that react according to the same external forcings, but differ by internal variability (as represented in that particular model). For our analysis, we calculated the global, annual mean surface temperature evolution for each member, and detrended them by subtracting the ensemble mean for each year, to end up with a time evolution that is solely due to internal variability. These time series were then separately added to the output from MAGICC6, to yield 32 new time series for each combination of climate forcer and mitigation scenario considered. Note, however, that when comparing two scenarios to calculate emergence, we shifted one time series by five years, so as to avoid comparing identical evolutions. This is realistic, as any substantial change in emissions would be comparable to a change in initial conditions, and therefore change the evolution of the simulated climate.

### Quantifying emergence

The final step is to define emergence in a quantitative way. In the real world, only one time evolution of global mean surface temperature will be realized, and it will be affected by both internal variability and a range of anthropogenic forcers. Hence, our constructed 32 member ensembles constitute a broad set of hypothetical futures that can be compared to a chosen baseline (the evolution following RCP4.5). Our starting point is a pair of time evolutions, including variability, from the baseline and an emission scenario. For this pair, we ask whether the full evolutions, from 2020 and up to a given year, are significantly different according to Student’s *t*-test (*p* < 0.05). We compare the time series of temperatures that would have been observed in each of these hypothetical futures, from the time when mitigation measures were implemented. The year when significance is first reached will vary between ensemble members. So, as our definition of emergence, we take the first year when at least 66% (21) of the baseline-scenario pairs are statistically significantly different.

In principle, the first 80 years of the CESM1 LENS simulations (1921–2000) could have been used to construct a second, independent set of 32 ensemble members for each perturbation. However, the two time periods differ significantly in their balance between GHG and aerosol forcing, which are generally assumed to influence modes of variability in different ways^40^. Hence, it is not trivially correct assume that the two time periods are equivalent in year-to-year variability and statistical distributions of e.g. ENSO occurrence and strength. For this reason, while we have confirmed that extending to 64 ensemble members in this way, or switching to the other set of members, has very little influence on the results presented, we choose to use only the original 32 members drawn from the continued evolution of RCP8.5.

## Supplementary information

Supplementary Information

## Data Availability

MAGICC6 is publicly available at live.magicc.org. CESM1 LENS simulations are available through http://www.cesm.ucar.edu/projects/community-projects/LENS/. The emission scenarios designed for the present study, and the corresponding output from MAGICC6, are available through Figshare (10.6084/m9.figshare.12366335.v1)

## References

[1] Bindoff N. L. et al. Detection and Attribution of Climate Change: from Global to Regional. in *Climate Change 2013: The Physical Science Basis. Contribution of Working Group I to the Fifth Assessment Report of the Intergovernmental Panel on Climate Change* (eds Stocker T. F. et al.). (Cambridge University Press, 2013).

[2] Myhre G. et al. Anthropogenic and Natural Radiative Forcing. in *Climate Change 2013: The Physical Science Basis. Contribution of Working Group I to the Fifth Assessment Report of the Intergovernmental Panel on Climate Change*.(eds Stocker et al.). (IPCC AR5 WG1, 2013).

[3] Allen M. R. et al. Framing and Context. in *Global Warming of 1.5* *°C. An IPCC Special Report on the impacts of global warming of 1.5* *°C above pre-industrial levels and related global greenhouse gas emission pathways, in the context of strengthening the global response to the threat of climate change, sustainable development, and efforts to eradicate poverty*. (IPCC, 2018).

[4] Kirtman B. et al. Near-term Climate Change: Projections and Predictability. in *Climate Change 2013: The Physical Science Basis. Contribution of Working Group I to the Fifth Assessment Report of the Intergovernmental Panel on Climate Change* (eds Stocker, T. F. et al.). (Cambridge University Press, 2013).

[5] Peters GP (2017). Towards real-time verification of CO2 emissions. Nat. Clim. Change.

[6] Medhaug I, Stolpe MB, Fischer EM, Knutti R (2017). Reconciling controversies about the ‘global warming hiatus’. Nature.

[7] Tebaldi C, Friedlingstein P (2013). Delayed detection of climate mitigation benefits due to climate inertia and variability. Proc. Natl Acad. Sci. USA.

[8] Marotzke J (2018). Quantifying the irreducible uncertainty in near‐term climate projections. Wiley Interdiscip. Rev.: Clim. Change.

[9] Harmsen JHM (2019). Long-term marginal abatement cost curves of non-CO2 greenhouse gases. Environ. Sci. Policy.

[10] IPCC. *Climate Change 2014: Mitigation of Climate Change. Contribution of Working Group III to the Fifth Assessment Report of the Intergovernmental Panel on Climate Change* (eds Edenhofer, O. et al.). (Cambridge University Press, Cambridge and New York, 2014).

[11] Meinshausen M, Raper SCB, Wigley TML (2011). Emulating coupled atmosphere-ocean and carbon cycle models with a simpler model, MAGICC6 – Part 1: Model description and calibration. Atmos. Chem. Phys..

[12] Kay JE (2015). The community earth system model (CESM) large ensemble project: a community resource for studying climate change in the presence of internal climate variability. Bull. Am. Meteorological Soc..

[13] Hawkins E, Sutton R (2012). Time of emergence of climate signals.. Geophys. Res. Lett..

[14] Frame D, Joshi M, Hawkins E, Harrington LJ, de Roiste M (2017). Population-based emergence of unfamiliar climates. Nat. Clim. Change.

[15] Frame DJ (2019). Emissions and emergence: a new index comparing relative contributions to climate change with relative climatic consequences. Environ. Res. Lett..

[16] Maher N (2019). The Max Planck Institute Grand Ensemble: enabling the exploration of climate system variability. J. Adv. Modeling Earth Syst..

[17] Hoesly RM (2018). Historical (1750–2014) anthropogenic emissions of reactive gases and aerosols from the Community Emissions Data System (CEDS). Geoscientific Model Dev..

[18] Shiogama H, Stone DA, Nagashima T, Nozawa T, Emori S (2013). On the linear additivity of climate forcing-response relationships at global and continental scales. Int. J. Climatol..

[19] Höglund-Isaksson L, Gómez-Sanabria A, Klimont Z, Rafaj P, Schöpp W (2020). Technical potentials and costs for reducing global anthropogenic methane emissions in the 2050 timeframe –results from the GAINS model. Environ. Res. Commun..

[20] Lund, M. T. et al. A continued role of short-lived climate forcers under the shared socioeconomic pathways. *Earth Syst. Dynam. Discuss*. 10.5194/esd-2020-9 (2020).

[21] IPCC. Climate Change and Land, An IPCC special report on climate change, desertification, land degradation, sustainable land management, food security, and greenhouse gas fluxes in terrestrial ecosystems. *Clim. Change Land*. https://www.ipcc.ch/srccl/ (2019).

[22] Samset BH (2016). Fast and slow precipitation responses to individual climate forcers: A PDRMIP multimodel study. Geophys. Res. Lett..

[23] Stjern CW (2017). Rapid adjustments cause weak surface temperature response to increased black carbon concentrations. J. Geophys. Res. Atmos..

[24] Samset BH (2018). Climate impacts from a removal of anthropogenic aerosol emissions. Geophys. Res. Lett..

[25] Samset BH, Lund MT, Bollasina M, Myhre G, Wilcox L (2019). Emerging Asian aerosol patterns. Nat. Geosci..

[26] Lund MT (2018). Short black carbon lifetime inferred from a global set of aircraft observations.. npj Clim. Atmos. Sci..

[27] Leach NJ (2018). Current level and rate of warming determine emissions budgets under ambitious mitigation. Nat. Geosci..

[28] Chalmers N, Highwood EJ, Hawkins E, Sutton R, Wilcox LJ (2012). Aerosol contribution to the rapid warming of near-term climate under RCP 2.6.. Geophys. Res. Lett..

[29] Shindell D, Smith CJ (2019). Climate and air-quality benefits of a realistic phase-out of fossil fuels. Nature.

[30] Shine KP, Fuglestvedt JS, Hailemariam K, Stuber N (2005). Alternatives to the global warming potential for comparing climate impacts of emissions of greenhouse gases. Climatic Change.

[31] Allen MR (2018). A solution to the misrepresentations of CO2-equivalent emissions of short-lived climate pollutants under ambitious mitigation.. npj Clim. Atmos. Sci..

[32] Smith CJ (2018). FAIR v1.3: a simple emissions-based impulse response and carbon cycle model. Geoscientific Model Dev..

[33] Deser C. et al. Insights from Earth system model initial-condition large ensembles and future prospects. *Nat. Clim. Change*. **10**, 277–286 (2020).

[34] Sutton R, Suckling E, Hawkins E (2015). What does global mean temperature tell us about local climate?. Philos. Trans. A Math. Phys. Eng. Sci..

[35] Arora VK (2013). Carbon–concentration and carbon–climate feedbacks in CMIP5 earth system models. J. Clim..

[36] van Vuuren DP (2011). The representative concentration pathways: an overview. Climatic Change.

[37] Li C (2017). India is overtaking China as the world’s largest emitter of anthropogenic sulfur dioxide. Sci. Rep..

[38] Collins M. et al. Long-term Climate Change: Projections, Commitments and Irreversibility. In *Climate Change 2013: The Physical Science Basis. Contribution of Working Group I to the Fifth Assessment Report of the Intergovernmental Panel on Climate Change* (eds Stocker T. F. et al.). (Cambridge University Press, 2013).

[39] Forster P. M., Maycock A. C., McKenna C. M. & Smith C. J. Latest climate models confirm need for urgent mitigation. *Nat. Clim. Change*. **10**, 7–10 (2019).

[40] Wilcox LJ, Highwood EJ, Dunstone NJ (2013). The influence of anthropogenic aerosol on multi-decadal variations of historical global climate.. Environ. Res. Lett..

