# Supplementary material for: Delayed emergence of a global temperature response after emission mitigation
Source: Nat Commun. 2020 Jul 7;11:3261. doi: 10.1038/s41467-020-17001-1 (PMC7341748; doi:10.1038/s41467-020-17001-1)
Supplement: Supplementary file 1 — Supplementary Information [file 41467_2020_17001_MOESM1_ESM.pdf]

Delayed emergence of a global temperature response after emission mitigation

Samset et al.

Supplementary materials

This supplement contains four additional figures that bolster the analysis presented in the main paper.

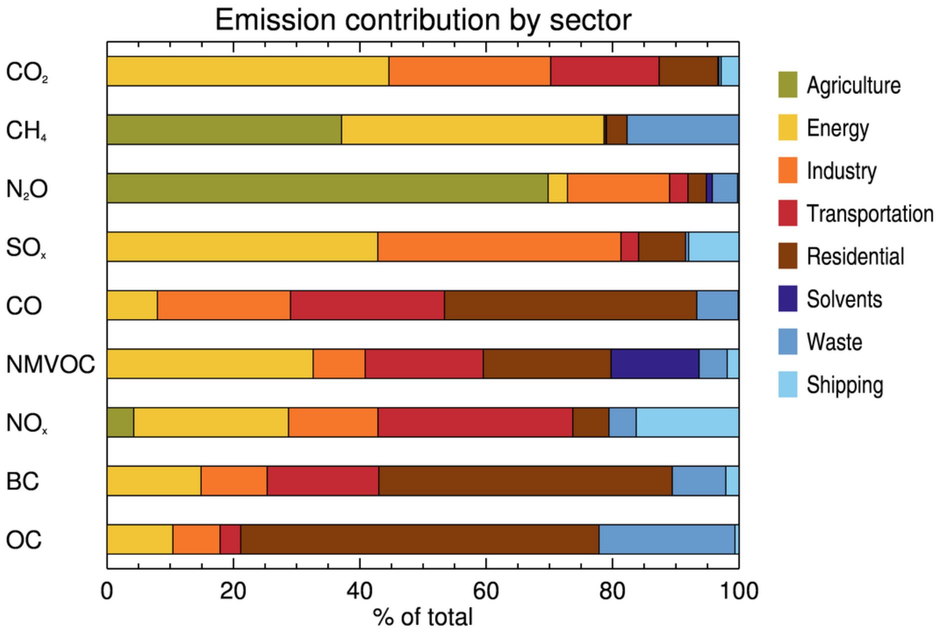

Supplementary Figure 1: Emission composition from the main global economic sectors, broken down into the components studied in this paper. All sectors are normalized to 100%, for ease of comparison. Figure based on 2014 emissions in the CEDS database[1].

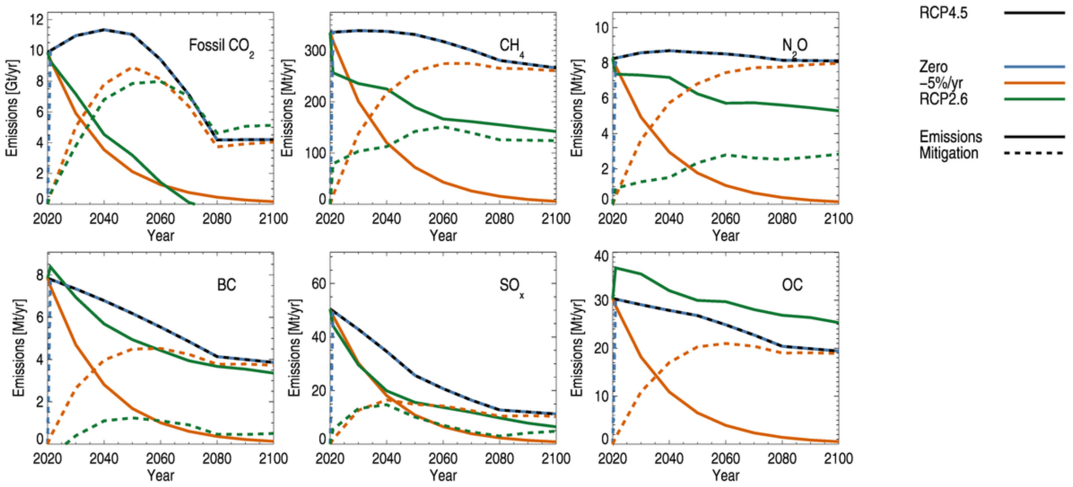

Supplementary Figure 2: Emission pathways used in the present paper. Absolute emissions (solid lines) and mitigated emissions (dashed lines) for all scenarios, for the major greenhouse gases and aerosol species. The mitigation lines show the difference between the baseline scenario (RCP4.5) and the scenarios with reduced emissions. (For the zero-emission case, the mitigation line (dashed blue) is identical to the emission line (solid black).)

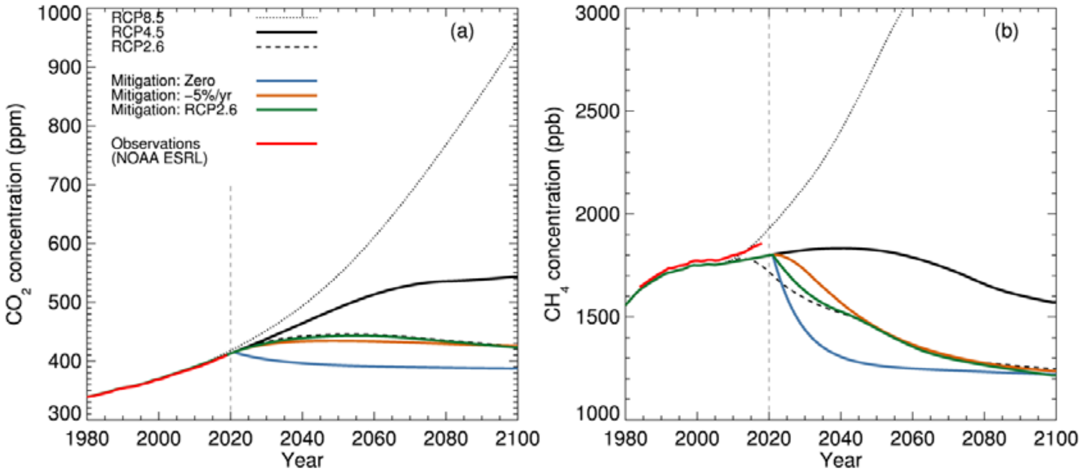

Supplementary Figure 3: Carbon cycle responses. Time evolution of atmospheric (a) CO_2 and (b) CH_4 concentrations, as simulated by MAGICC6[2] and its representation of the carbon cycle, for the core scenarios used in the present paper. The red lines show observations from NOAA ESRL[3].

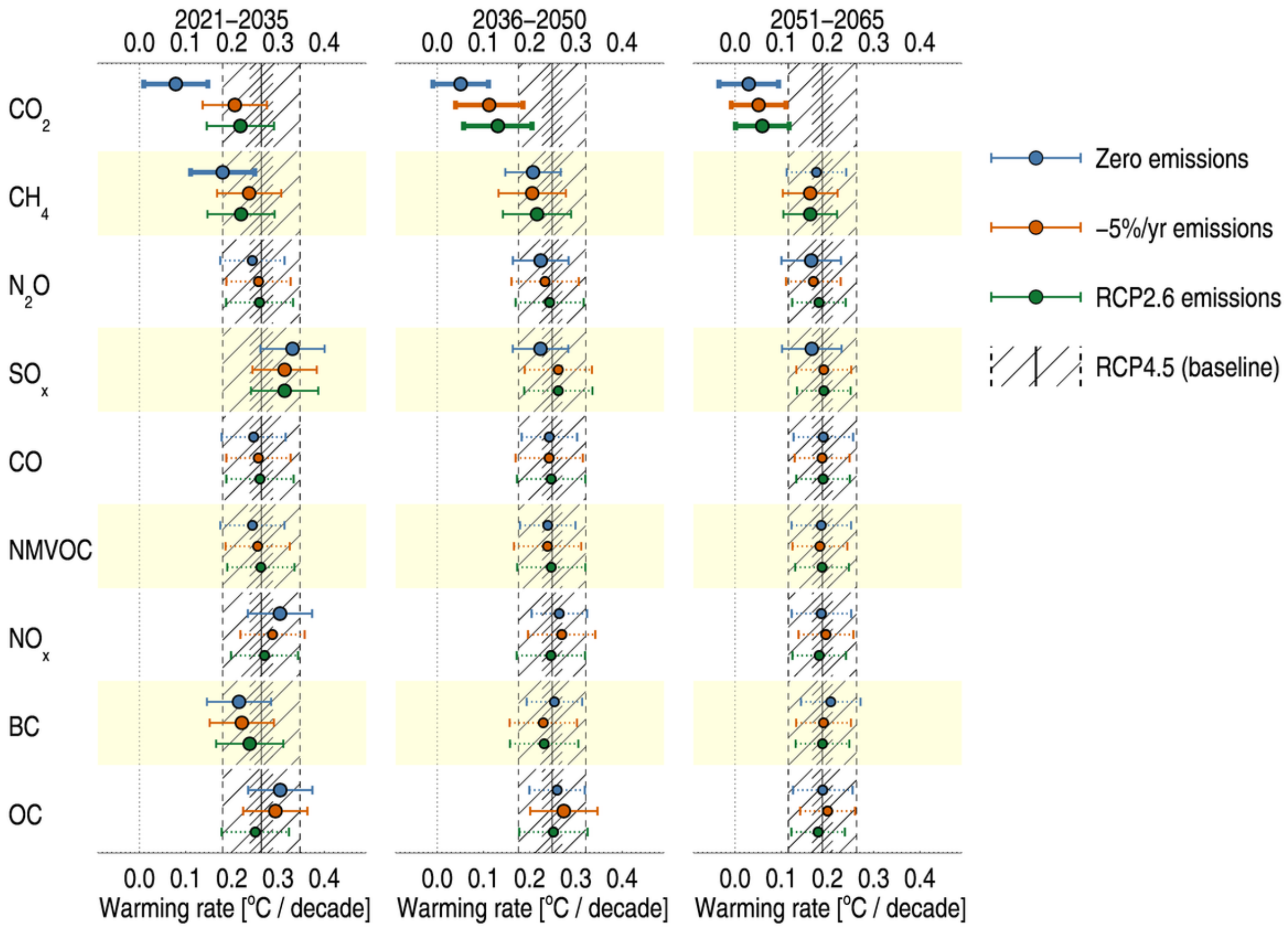

Supplementary Figure 4: Warming rates. As the lower part of Figure 4, but for trends of 15 years.

Supplementary references

1 Hoesly, R. M. *et al.* Historical (1750–2014) anthropogenic emissions of reactive gases and aerosols from the Community Emissions Data System (CEDS). *Geoscientific Model Development* **11**, 369-408, doi:10.5194/gmd-11-369-2018 (2018).

2 Meinshausen, M., Raper, S. C. B. & Wigley, T. M. L. Emulating coupled atmosphere-ocean and carbon cycle models with a simpler model, MAGICC6 – Part 1: Model description and calibration. *Atmospheric Chemistry and Physics* **11**, 1417-1456, doi:10.5194/acp-11-1417-2011 (2011).

3 Dlugokencky, E. & Tans, P. *NOAA/ESRL* (2020).
